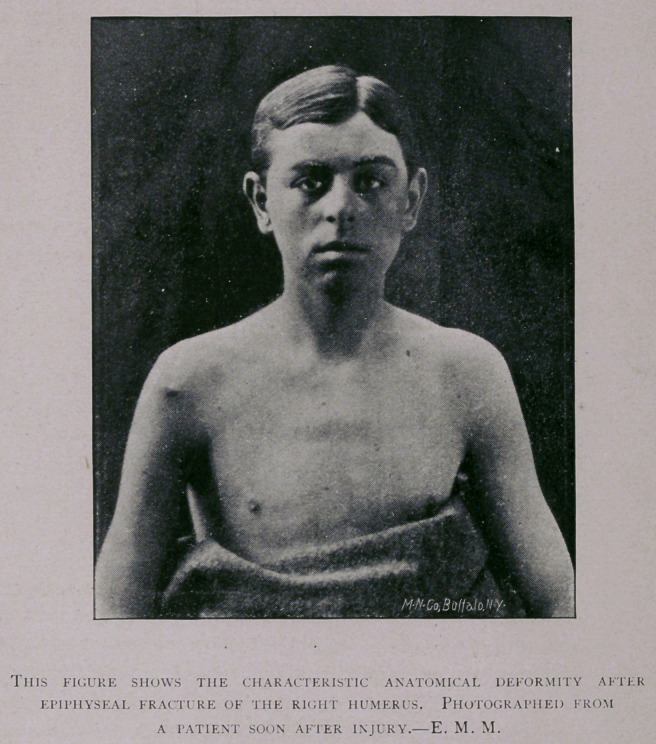# The Surgery of the Upper Extremities

**Published:** 1889-03

**Authors:** Edward M. Moore

**Affiliations:** No. 74 South Fitzhugh Street; Rochester, N. Y.


Buffalo Medical and Surgical Journal

Vol. XXVIII. MARCH, 1889. No. 8

### Original Communications.

### *THE SURGERY OF THE UPPER EXTREMITIES.*

Part II.

### EPIPHYSEAL FRACTURE OF THE SUPERIOR EXTREMITY OF THE HUMERUS.[1]

By EDWARD M. MOORE, M. D., Rochester, N. Y.

Professor R. W. Smith, in his admirable treatise on fractures in the neighborhood of joints, opens the subject of his paper with the following statement:

" Fracture of the humerus through the line of its superior epiphysis is an accident which not unfrequently occurs in early life. It is attended by a considerable degree of deformity, but of so striking a nature, that there is no great difficulty in recognizing the true nature of the injury.

" The axis of the arm is directed from above, within and before, downwards, outwards and backwards; the elbow, however, projects but little from the side, and can be brought into contact with it with facility; the head of the bone can be distinctly felt in the glenoid cavity; a slight depression is seen beneath it, and it remains motionless, when the shaft of the humerus is rotated.

"The most remarkable feature, however, of this injury is a striking and abrupt projection, situated beneath the coracoid process, and caused by the upper extremity of the lower fragment or the shaft of the bone, drawn inwards by the muscles, which constitute the folds of the axilla; there is but little displacement as regards the length of the bone, for the extremity of the inferior fragment is seldom drawn so far inwards as to enable it to clear completely the surface of the superior. Were this to occur, the humerus would, of course, be drawn upwards by the muscles passing from the shoulder to the arm, in a direction parallel, or nearly so, to the axis of the humerus, and a corresponding diminution in the length of the limb would result.

1. Portion of an address before the Central New York Medical Association, November 20, 1888. Continued from page 369.

"This remarkable and abrupt projection does not present the sharp, irregular margin of an ordinary fracture; on the contrary, it feels rounded, and its superior surface is smooth and slightly convex. The latter can be felt as plainly as the cup-like cavity of the head of the radius, in cases of luxation of that bone backwards, at the elbow-joint. By pressing the upper end of the lower fragment outwards, and directing the elbow inwards during extension and counter-extension, crepitus can be perceived, and the deformity removed without much difficulty; but the moment the parts are abandoned to the uncontrollable action of the muscles, the deformity recurs."

I quote this with reference to diagnosis, and a short summary at the latter part of his paper, as follows:

"The chief diagnostic signs of the separation of the superior epiphysis of the humerus are an abrupt projection beneath the coracoid process, caused by the upper end of the lower fragment, and the immediate recurrence of the deformity when the means employed for its reduction cease to be in operation."

Also, "There is no fracture incidental to the upper end of the humerus, in which it is more difficult to maintain the fragments in their proper relative position."

But, notwithstanding the very positive statements with reference to the diagnosis, by the author as quoted, great confusion has existed, and still exists, as regards the exact nature of fracture of the humerus at the point under discussion. Nevertheless, after the very clear statement of symptoms that has been given by Sir A. Cooper, Prof. R. W. Smith, and Prof. Frank Hamilton, one would hardly suppose that error of diagnosis would occur. But it does occur, and constantly. I think this results from the fact that a clear conception of the change of position has not been put forth, and more than this, and growing out of it, no method that secures reduction of the fracture has thus far, according to my knowledge, been proposed. If there had been a good method, especially if it were connected with its rationale, the experimentum crucis would have been present to clear up doubt of diagnosis, as well as rectify deformity. We must, therefore, look to the configuration of the epiphyseal junction, to explain the position of the bones after the separation. Taking the head of a bone from a subject ten years of age, parted by maceration, we find the angle made by the junction of the plane projected through the anatomical neck, (and which makes about two-fifths of the whole surface,) with the plane passing below the tuberosities, measures about 100 degrees.

"The development of the humerus," says Gray, "is by one ossific point for the shaft, one for the head, and one for the greater tuberosity. At birth, the shaft is ossified nearly in its whole length, the extremities remaining cartilaginous. Between the first and second years, the center for the tuberosities makes its appearance, usually by a single ossific point, sometimes another for the lesser tuberosity, which does not appear until after the fourth year. By the fifth year, the centers for the head and tuberosities have enlarged and become joined, so as to form a single large epiphysis."

The constancy of the symptoms makes this fracture to differ from most that occur. The projection of the angle of the diaphysis, making the singular appearance about one or two inches below the acromion, according to the age of the patient, and the shortening of the humerus from half to three-fourths of an inch, will render the diagnosis so plain that the difficulty should not be mistaken for any other injury. But it is constantly mistaken, and the mistake is acted on; severe and protracted extensions being made to reduce reduction.

A little irregularity is found on the surface, but the plane is pretty accurate. On the surface of the head is found a depression, into which, if the shaft is moved inward about one-fourth of its breadth, there is a coaptation, which arrests the movement of the bones, and which also fixes the shaft at the point which Smith and other observers have described. The change of coaptation in the facettes explains the constancy of the symptoms, so regularly noticed, by which the projection forward of the diaphysis replaces the rotundity of the head. This change has a little analogy to dislocation of the knee laterally, by which the extreme condyle of the femur is jumped, as it were, into the cup on the internal condyle of the tibia, and retained by the tension of muscles. The posterior facette of the diaphysis, by the force of the blow and action of the muscles, becomes fitted to the anterior facette of the head. Of course, in such injuries as fractures, the muscles are apt to acquire their greatest tension at the moment of separation. Those that act in the direction of the shaft press upward. The head is set upon the shaft at an angle, and if there were no other cause, they would have a tendency to roll the now movable head upon the glenoid surface. But, besides the muscles parallel with the shaft, there are those attached to the tubercles, whose traction would now roll the head so as to produce dislocation of the facettes. This position throws the superior edge of the diaphysis forward, and then retains it there; for the universal testimony asserts the return to the same place, even after the strongest extension. It is the observation of all, that traction will cause the deformity to disappear. That traction does not produce reduction, is also equally evident, for the reason that it does not remain in place after it appears to be reduced. I think, however, that the relief of the deformity by traction is not as great as observers have thought. When extension is made, the head will undoubtedly work on the projecting angle of the diaphysis, and the tense deltoid, pressing the shaft back, would restore the rotundity of the shoulder. But the reduction of the luxated surface does not take place, and the cessation of the extension finds the capsular muscles ready to roll the head over and project the diaphysis forward. In consequence of the very common error of diagnosis, and the belief that a luxation is present, the attempt at reduction is made by extension. The evidence of the mistake in supposing that restoration had occurred after extension, is quite complete, for no one asserts that the appearance of reduction is permanent, the symptoms recurring as soon as the extension ceases.

In view of such a displacement, the natural mode of reduction would seem to be one that would carry the diaphysis backward, and thus restore the corresponding facettes to their normal

Explanation of Plate.—Figs. 1 and 2 represent the head and part of the shaft of the humerus, from a boy ten years of age, and are photographed life-size. These have been separated by maceration, and replaced. Fig. 1 gives a profile of the bone regarded from its external aspect. Fig. 2 gives a profile of the same bone regarded from its internal aspect. The dotted line A indicates the anatomical neck. The epiphyseal line B is seen to correspond with the anatomical neck along nearly one-half of its length, and diverges nearly at a right angle below the tuberosities. Fig. 3 represents the same bone in the position it retains after fracture along the epiphyseal line. The smaller facette of the superior diaphyseal surface is locked with the larger facette of the corresponding surface of the head.

Fig. 1.

Fig. 2.

Fig. 3.

positions. This I succeeded in doing, not by extension, but by carrying the humerus *forward* and *upward.* The head will roll upon the glenoid surface in any motion of the arm, until restrained by its capsule. While the humerus is still back of the central line of the body, the head is rolled upward, and long before the humerus is brought up perpendicularly, the capsule at the lower border of the head has become tense, thus holding it firm while the humerus, being drawn up and restrained by its muscles, slides the diaphysis backward, producing a coaptation of the corresponding facettes. If these facettes have changed their position, or rather if one is entirely thrown forward, and the internal facette of the diaphysis is brought and retained in contact with the external one of the head, it must be obvious that when the shaft is hanging perpendicularly, the head must be rolled in such a position that the arm, if there were no fracture, would be nearly at right angles with the body. Of course, the head will roll around in any direction, in consequence of the lock of the surfaces, unrestrained by any influence except the capsule. By a firm grasp of the thumb on one side, and the fingers on the other, the head may be so far restrained as to procure crepitus. But the hold is not secure enough to answer the necessities of reduction. Yet, when the head is rolled over until the capsule becomes tense, the restraint will become perfect, if the force be applied in a direct line.

A very slight extension of the humerus will *now* preserve the relation of the two surfaces. While this moderate extension is maintained, the arm can be brought down to the side, all deformity having disappeared. In short, a reduction of the fracture has been accomplished.

From these statements, it becomes apparent that this fracture possesses many of the characters of a luxation. The bones after separation are moved into, and retained in, a definite place in obedience to definite forces.

A fracture in other than an epiphyseal line is, as a matter of course, irregular at its surfaces; and while we may find that a general law may govern fractures at certain points, we also find frequent variations incidental to the form of the surfaces. No better illustration of this truth can be found than in the fractures immediately below, that is to say, at the surgical neck; for in these it is well known that the usual displacement is that of the shaft inward, in obedience to the pectoralis major and latissimus dorsi. But, even in this fracture, there are numerous cases where the obliquity of the surfaces has caused a reversal of the usual relation.

The admission is made by all that no plan of treatment that has been heretofore proposed is likely to succeed; that the characteristic deformity appears upon the removal of dressings—but I have found no difficulty whatever in the use of Swinburne's extension plan. The axillary border has been sufficiently firm to bear the extension necessary, and the axillary band, by passing over the head of the humerus, assists in extension. I find no pressure sufficiently great to disturb the axillary nerves. Indeed, the method leaves nothing to be desired, and the facility with which it may be applied and worn renders it quite perfect. In every case, it has entirely succeeded. However, I think it can do this only when reduction has occurred.

The following cases confirm the correctness of the methods, as well as illustrate the rationale of displacement:

John Duff, æt. 14; September, 1868. Fell from a loaded wagon, striking on the right shoulder. He was seen two hours after the accident by a well-instructed physician, who committed the error of diagnosis formerly alluded to, in regarding the case as one of dislocation. Violent traction must have been made, for two men pulled at the arm; but when the extension ceased, the deformity reappeared, although the supposed luxation was believed to have been reduced. This having failed, it was renewed the next morning, in consultation with another physician. The patient was then sent to me the same day. The characteristic symptoms were present, as already detailed. It is also to be noted that on rotating the arm before reduction, there was no crepitus until the head was grasped firmly. The bandage and splint were worn during the period of four weeks. The restoration was absolutely perfect, and so remains, as proved by a recent examination.

Nelly Coates, æt. 16. Fell from a height of about six feet, striking on the shoulder in front. The patient was seen by the family physician immediately, but he did not recognize any displacement. Fourteen days later she was seen by another physician of great experience, but, on examination, he also thought there was no displacement. The patient still suffering, a third physician was called, who thought there was luxation. I saw the case seventeen days after the accident. The symptoms, as detailed in the *résumé*, were all present. It was treated by first reducing the displacement, and the application of Swinburne's dressing, which was worn about two weeks. A year afterwards, the arm was examined, and the result was so perfect that no difference in form or motion could be discovered.

Charles Bunnel, æt. 6 years; on March 15, 1873, fell, as it was thought, on his shoulder. He was seen by Dr. C. Hammond, of Monroe county, on the day following, who diagnosticated luxation, but, on making further examination while the patient was under the influence of ether, "found the movements of the shoulder perfect;" and getting no crepitus, supposed the deformity resulted from ecchymosis. At the end of two weeks, the swelling had subsided, and the prominence was more marked. At this time, (March 18, 1873,) I saw the patient for the first time. The boy was the unfortunate subject of infantile paralysis, which had occurred between the first and second year, and affected the arm that was broken. My views had been pretty well settled before, but now I had a case which, though not quite, was almost as capable of demonstrating the facts as an autopsy. I need not remind the profession of the shrunk muscles and lax skin that invest the bone in such cases. As is usual, there was some muscular power; but while in the strong and healthy there is much diminution of utility in the arm, in this patient the little he possessed was gone, and hence its restoration became a matter of unusual interest. The adhesion of the bones was readily broken up by carrying the arm upward. The muffled crepitus was obvious, and the restoration perfect. Three months afterward, Dr. Hammond, in a note to me, remarks that the "restoration is complete in every respect."

RÉSUMÉ.—1st. The symptoms of this fracture are striking and uniform. The shaft of the humerus is so inclined as to carry the elbow a little backward and outward, while the superior end of the shaft is brought forward, so as to make a prominence less rounded than the head and lower down. This is usually found about an inch and a half below the acromion (the distance varying a little with the size of the youth) and near the coracoid process. The curved line from the acromion down to this projection has a long sweep, instead of the small sphere of the natural head. This appearance is pathognomonic, and may be safely trusted in diagnosis, without insisting upon crepitus. As in other epiphyseal fractures, this is not clear and sharp as when the fracture is of bone, but is muffled. When the arm is moved gently, and without grasping the head, the peculiar lock of the surfaces is sufficient to cause the head to rotate, and thus the timid practitioner fails in getting his pathognomonic sign—but if the head be firmly grasped, it cannot only be felt in the glenoid cavity, but be held sufficiently firm to get this muffled crepitus by rotating the humerus, or by carrying the elbow inward and thus rubbing the two surfaces on each other. In addition to these striking symptoms, we may add the fact of a shortening of half an inch or a little more in the length of the humerus. When the two shoulders are inspected from behind, the impression produced on the mind of the surgeon is that described as subluxation, for there is a slight flattening of the shoulder. The breadth of the shoulder is also increased when seen in profile. The motions of the arm are somewhat circumscribed. The ability to carry it upward and forward as well as upward and outward, is impossible much beyond a right angle with the body.

2d. The reduction of the fracture is effected by carrying the arm forward and upward to the perpendicular line.

3d. The retention is effected by moderate extension, while bringing the arm down to the side, maintaining the slight extension until dressings for the purpose of continuing it are applied. Swinburne's method fulfills the indication easily and perfectly.

4th. Even if not restored, the arm soon becomes useful, and nature gradually rounds off the prominence of the diaphysis, and elongates the capsules at the lower border, allowing the motions upwards to improve.

In 1874, I presented this subject, mainly as in the preceding pages, before the American Medical Association; since that time,

THIS FIGURE SHOWS THE CHARACTERISTIC ANATOMICAL DEFORMITY AFTER EPIPHYSEAL FRACTURE OF THE RIGHT HUMERUS. PHOTOGRAPHED FROM A PATIENT SOON AFTER INJURY.—E. M. M.

many cases have been brought me. They only confirm the correctness of the diagnosis and treatment of this difficulty. I do not recollect a single case in which the diagnosis was made out by the attending surgeon. When I read the paper before the New York County Society, such distinguished men as Gurdon Buck, Prof. Post and Dr. Little, confessed that they had never seen a case of this fracture during their lives. They had undoubtedly seen them, but had overlooked them under the impression that the swelling was due to blood-clots. But when the fracture has been once recognized, it cannot be confounded with any other injury. It has its special diagnostic marks. There is a condition that I am sure Prof. Robert Smith has not recognized. There is a difference in the sharp definition of the projection of the anterior border of the upper end of the diaphysis. This seems to be due to the fact that sometimes the point of the bone has passed through the fibres of the deltoid, and at other times it is covered by the muscle. Those cases that seem to present the point directly under the skin are much more rare than those which are covered by muscle. It is the latter condition that is especially overlooked.

No. 74 South Fitzhugh Street.

## Figures and Tables

**Fig. 1. f1:**
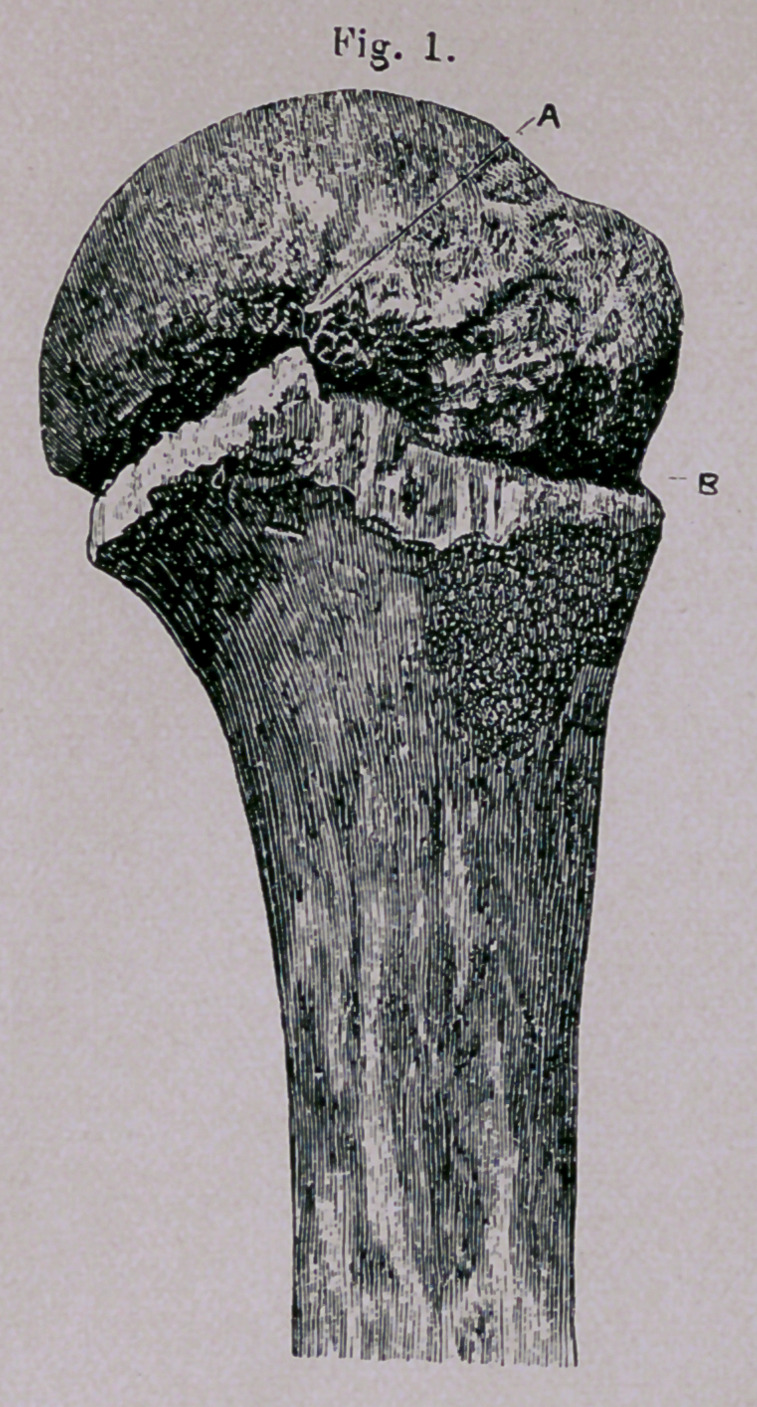


**Fig. 2. f2:**
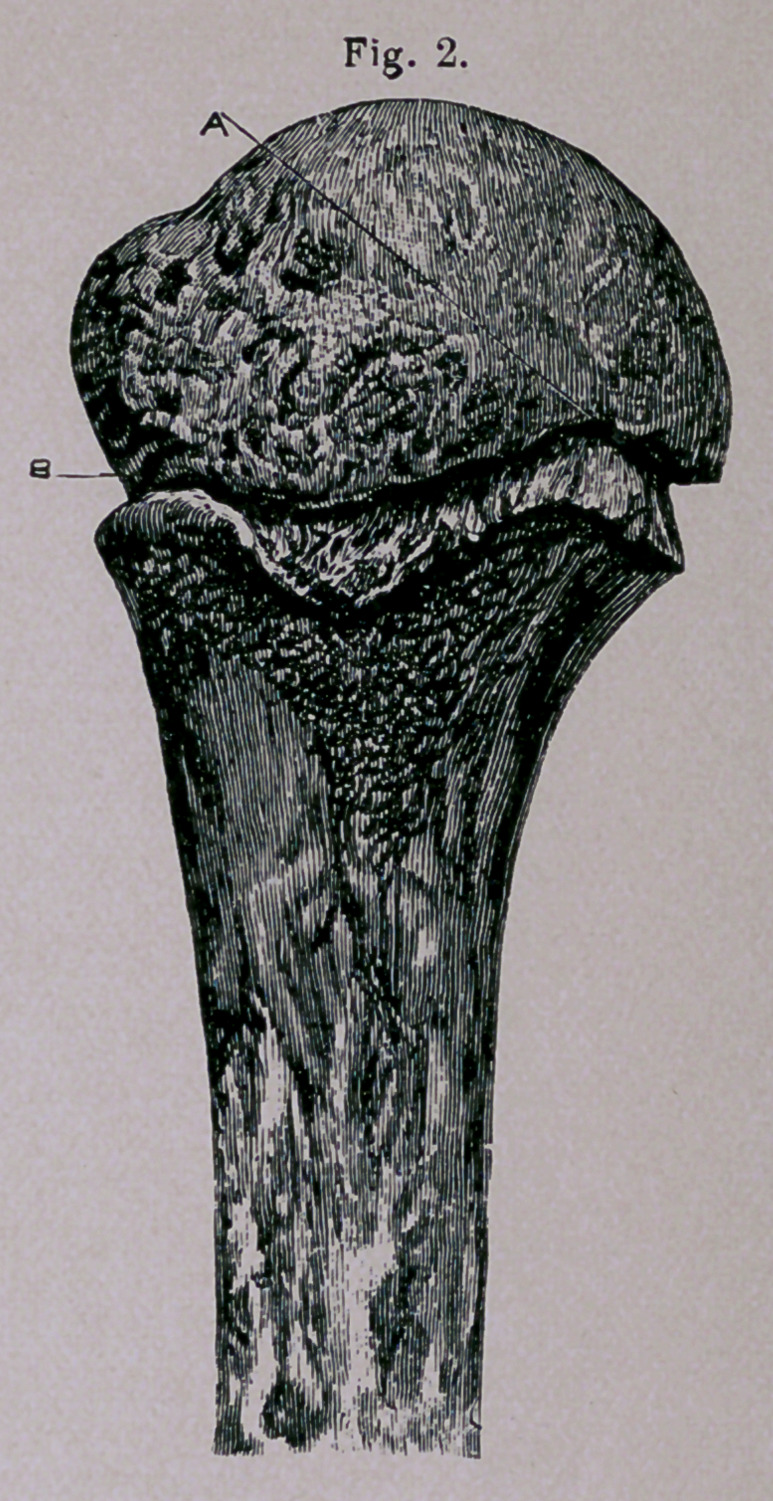


**Fig. 3. f3:**
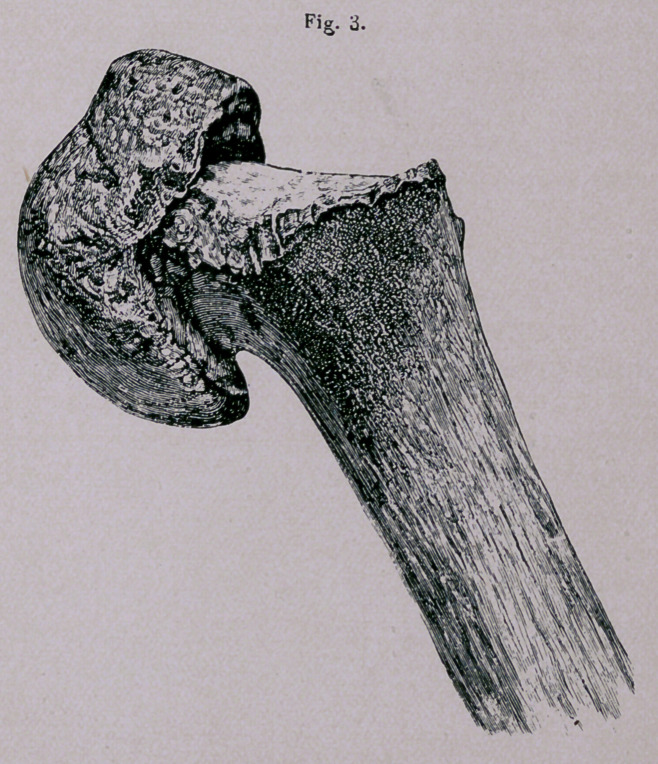


**Figure f4:**